# Galectin-13, a different prototype galectin, does not bind β-galacto-sides and forms dimers via intermolecular disulfide bridges between Cys-136 and Cys-138

**DOI:** 10.1038/s41598-018-19465-0

**Published:** 2018-01-17

**Authors:** Jiyong Su, Yue Wang, Yunlong Si, Jin Gao, Chenyang Song, Linlin Cui, Runjie Wu, Guihua Tai, Yifa Zhou

**Affiliations:** Jilin Province Key Laboratory for Chemistry and Biology of Natural Drugs in Changbai Mountain, School of Life Sciences, Northeast Normal University, Changchun, 130024 PR China

## Abstract

During pregnancy, placental protein-13 (galectin-13) is highly expressed in the placenta and fetal tissue, and less so in maternal serum that is related to pre-eclampsia. To understand galectin-13 function at the molecular level, we solved its crystal structure and discovered that its dimer is stabilized by two disulfide bridges between Cys136 and Cys138 and six hydrogen bonds involving Val135, Val137, and Gln139. Native PAGE and gel filtration demonstrate that this is not a crystallization artifact because dimers also form in solution. Our biochemical studies indicate that galectin-13 ligand binding specificity is different from that of other galectins in that it does not bind β-galactosides. This is partly explained by the presence of Arg53 rather than His53 at the bottom of the carbohydrate binding site in a position that is crucial for interactions with β-galactosides. Mutating Arg53 to histidine does not re-establish normal β-galactoside binding, but rather traps cryoprotectant glycerol molecules within the ligand binding site in crystals of the R53H mutant. Moreover, unlike most other galectins, we also found that GFP-tagged galectin-13 is localized within the nucleus of HeLa and 293 T cells. Overall, galectin-13 appears to be a new type of prototype galectin with distinct properties.

## Introduction

Galectins, widely expressed by cells within the animal kingdom^1^, all have at least one evolutionarily conserved carbohydrate recognition domain (CRD) that can bind β-galactosides^1,2^. Based on differences in their overall structures, galectins are classified into three types: prototype (galectin-1, -2, -5, -7, -10, -11, -13, -14, -15, and -16), chimera (galectin-3), and tandem-repeat (galectin-4, -6, -8, -9, and -12)^2–4^. Functionally, galectins mediate a variety of biological processes, including cell differentiation, adhesion, and apoptosis^5,6^, and they also have impacts on innate and adaptive immunity as they are key determinants of acute and chronic inflammation, immune tolerance, and host-pathogen interactions^4,7–9^.

Galectin-13 (Gal-13, a.k.a. placental protein 13 or PP13) was first isolated from human placenta^10,11^, and its gene has been mapped to chromosome 19q13.1, which is in close proximity to the galectin-4, -7, -10 and 14^12^ genes. Gal-13 exhibits approximately 54% amino acid identity and 56% nucleic acid identity with galectin-10^12–14^. As with other galectins, Gal-13 is synthesized in the cytoplasm and can be externalized to the extracellular matrix via a non-classical transport pathway^15^. Bohn and Than *et al*. found that this lectin is a 32 kDa homodimer, whose monomer subunits are linked by disulfide bonds^10–12,14^. However, the cysteine residue positions that form those disulfide bridges are unknown. Structural modeling of lectin based on the “jelly-roll” fold common to other galectins showed that it consists of five- and six-stranded β-sheets of the CRD S-face and F-face, respectively^16^.

Even though Gal-13 is predominantly expressed in the placenta (specifically in the syncytiotrophoblast)^12^, it is also expressed in the spleen, kidney, and bladder, as well as in liver adenocarcinoma, neurogenic tumors, and malignant melanoma^14^. This lectin can also induce apoptosis of activated T cells and regulate immune tolerance between maternal and fetal tissues^13^. During pregnancy, Gal-13 levels in maternal serum continuously increase, and near term, its concentration is approximately three times higher than in the serum of non-pregnant women. Post-partum, Gal-13 vanishes from maternal blood within 2–5 weeks^17^. In pregnant rats, Gal-13 can regulate blood pressure and expand utero-placental vasculature. Overall, Gal-13 plays a key role in pregnancy and could be valuable at discriminating aneuploid from euploid pregnancies^18^ and other obstetrical complications, including intrauterine growth restriction (IUGR), early IUGR, and IUGR associated with pre-eclampsia^19,20^. A reduced Gal-13 mRNA level in placentas from the first and third trimesters has been assumed to indicate HELLP syndrome and pre-eclampsia^21,22^. Therefore, the Gal-13 concentration in maternal serum may be useful for predicting pre-eclampsia^23^. Some reports indicate that the predictive accuracy increases when Gal-13 measurements are combined with second-trimester uterine artery Doppler measurements^19,20^, whereas another report dispels the claim^24^. The measurement of serum Gal-13 at 11–13 weeks does not enhance the screening accuracy for early pre-eclampsia^25,26^. Recently, screening a human cDNA library revealed a novel variant (DelT221) of Gal-13 that encodes for a shorter polypeptide of 110 amino acids compared to the 139 amino acids found for the wild-type lectin^27^. The absence of some C-terminal residues in this variant causes the lectin to lose its ability to induce T-cell and macrophage apoptosis^13,27^. Women having this variant display a greater incidence of severe pre-eclampsia^13,27^.

Despite extensive physical and biochemical studies on Gal-13, its three-dimensional structure is unknown. The present study focuses on gaining a better understanding of Gal-13 structure-function relationships. Here, we report the crystal structure of Gal-13, as well as biochemical and cellular studies on ligand binding specificity and distribution of the lectin in cells.

## Results

### Crystal structure of Gal-13

**Figure 1:**
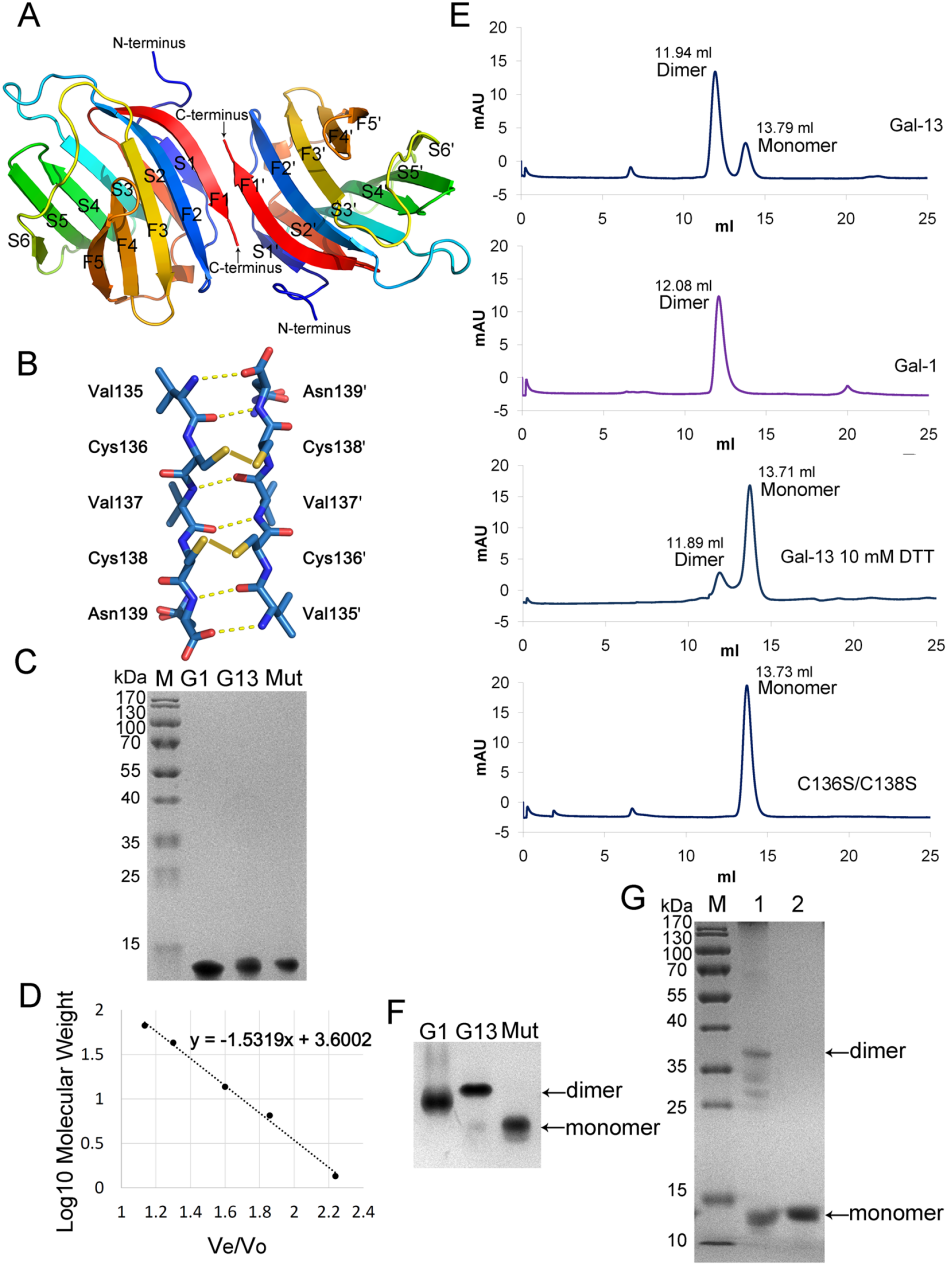
Gal-13 structure. (**A**) The overall crystal structure of Gal-13. Gal-13 was crystallized as a homodimer with only one molecule in the asymmetric unit. Each Gal-13 monomer contains 11 β-strands (S1-S6, F1-F5). The dimer interface is located between the F1 and F1′ strands. (**B**) The last five residues (Val135, Cys136, Val137, Cys138 and Asn139) within the F1 strands form the interface. Cys136 forms a disulfide bond with Cys138′ from the opposing Gal-13 monomer subunit. Similarly, Cys138 forms a disulfide bond with Cys136′ from another Gal-13 monomer subunit. Val135, Val137 and Asn139 from two Gal-13 monomers form 6 hydrogen bonds. The disulfide bonds are indicated by yellow lines. The hydrogen bonds are indicated by yellow dashed lines. The electron density of the two disulfide bonds is presented in Fig. S1. (**C**) Normal SDS-PAGE of Gal-1, Gal-13 and the double-mutant C136S/C138S. “M” indicates the protein molecular weight marker. “G1” indicates Gal-1, “G13” indicates Gal-13, and “Mut” indicates the C136S/C138S mutant. The original full-length gel is presented in Fig. S2. (**D**) Four known proteins and a compound were used to generated a standards equation (y = −1.5319x + 3.6002) for calculating Gal-1 and Gal-13 molecular weights. Ve indicates the protein elution volume. Vo indicates the void volume of column determined by thyroglobulin (669.0 kDa). (**E**) Gel filtration of Gal-1, Gal-13 and the double-mutation variant (C136S/C138S). The main elution peak of Gal-13 is similar to that for Gal-1, indicating that Gal-13 mainly forms dimers. A small Gal-13 peak corresponding to the monomer state is located behind the main peak. The mutant C136S/C138S only exists as a monomer. The gel filtration profile shows Gal-13 could form a dimer even in the presence of 10 mM DTT. (**F**) Native PAGE of Gal-1, Gal-13 and C136S/C138S. Wild-type Gal-13 mainly forms dimers, however, it could also exist as a monomer. The double-mutant C136S/C138S exists only as a monomer. The original full-length gel is presented in Fig. S3. (G) SDS-PAGE of Gal-13 with or without DTT reduction. M indicates the protein molecular weight marker. Without DTT, Gal-13 could form a dimer (see lane 1). The original full-length gel is presented in Fig. S4.

We crystallized wild-type Gal-13 and its R53H mutant using two different conditions. The wild-type lectin was crystallized in a solution containing PEG 4000. Using the same conditions, however, we were unable to crystallize the R53H variant and had to use a different solution containing PEG 3350. With one molecule per asymmetric unit, Matthew’s coefficient was calculated to be 2.07 Å^3^/Da, corresponding to a solvent content of 40.5%. Although crystallization conditions were different, the space group for the crystals was the same (Table 1). As with all other galectins, Gal-13 adopts the typical “jelly-roll” fold formed by sandwiching two anti-parallel β-sheets that consist of six (S1-S6) and five (F1-F5) β-strands and a short α-helix connecting strands S2 and F5 (Fig. 1A). Superposition of the structures of wild type Gal-13 and R53H shows that they are essentially the same with a low Cα RMSD value of 0.122.

**Table 1:** Data collection and refinement statistics.

Parameters	Gal-13	R53H-Glycerol	R53H
PDB code	5XG7	5XG8	5Y03
Resolution (Å)	19.08–1.55 (1.58–1.55)	19.09–1.55 (1.58–1.55)	19.05–2.12 (2.18–2.12)
Space group	C222	C222	C222
Unit cell parameters (a,b,c) (Å)	57.88 92.10 50.75	58.04 92.21 50.70	57.88 92.31 50.63
No. of measured reflections	128985 (5098)	123852 (4466)	15072 (1237)
No. of unique reflections	19963 (926)	20107 (983)	7985 (647)
Completeness (%)	99.5% (95.3%)	99.9% (99.8)	99.8 (99.6)
Multiplicity	6.5 (5.5)	6.2 (4.5)	1.9 (1.9)
*R*_merge_ (%)	8.1 (25.9)	4.6 (16.4)	10.0 (25.9)
<*I*/δ(*I*)>	13.6 (5.0)	18.3 (5.4)	6.6 (4.5)
*R*_model_ (%)	15.94	17.1	17.54
*R*_free_ (%)	18.73	19.46	22.17
Rmsd bond lengths (Å)	0.009	0.005	0.008
Rmsd bond angles (°)	1.066	0.82	0.978
Ramachandran plot^f^ residues in favored regions (%)	95.6	96.3	96.3
Ramachandran outliers (%)	0.7	0.7	0.7

Gal-13 crystallizes as a dimer with its subunit interface formed by the last five C-terminal residues (Val135, Cys136, Val137, Cys138, and Asn139) that are located within the F1 β-strand. Aside from the two intermolecular disulfide bonds (Fig. 1B, Fig. S1), residues Val135, Val137 and Asn139 at the interface form six hydrogen bonds that further stabilize the dimer structure. The amide hydrogen of Val135 forms a hydrogen bond with an oxygen atom from the terminal carboxylate group, the carbonyl oxygen of Val135 forms a hydrogen bond with the main chain amide hydrogen of Asn139, and the amide hydrogen of Val137 forms a hydrogen bond with the carbonyl oxygen of Val137 from the opposing Gal-13 dimer subunit (Fig. 1B). Because Gal-13 forms a homodimer with twofold symmetry, the set of residues Val135, Val137, and Asn139 forms a total of six hydrogen bonds.

On a normal SDS-PAGE, the band for Gal-13 (with reducing agent and boiling) runs higher than that of Gal-1, in line with the difference in their molecular weights (i.e., 16.1 kDa for Gal-13 vis-à-vis 14.7 kDa for Gal-1) (Fig. 1C, Fig. S2). We characterized the multimeric states of Gal-13 by size exclusion chromatography. Using the elution volumes of four standard proteins and one chemical compound (bovine serum albumin, ovalbumin, ribonuclease A, aprotinin, and vitamin B12), we calculated the apparent molecular weights of our galectin samples (Fig. 1D). For Gal-13, we observe two elution peaks, one at 11.94 ml (calculated to 28.1 kDa) and the other at 13.79 ml (calculated to 13.0 kDa). The major elution peak for Gal-13 occurs at essentially the same position as that for dimeric Gal-1 (12.08 ml, calculated to 26.5 kDa) (Fig. 1E). Furthermore, in the presence of 10 mM DTT, Gal-13 elutes from the gel mostly as a monomer (13.71 ml, calculated to 13.5 kDa) with a small amount of dimer (11.89 ml, calculated to 28.7 kDa), indicating that Gal-13 indeed forms dimers that are stabilized by disulfide bonds. As further proof of this finding, we produced the double-mutant C136S/C138S, which elutes as a monomer in gel filtration (13.73 ml, calculated to 13.4 kDa, Fig. 1E, Fig. S3). In normal SDS-PAGE, the band for Gal-13 (with reducing agent and boiling) runs higher than that for Gal-1, in line with the difference in their molecular weights (i.e., 16.1 kDa for Gal-13 vis-à-vis 14.7 kDa for Gal-1) (Fig. 1C, Fig. S2). Native PAGE demonstrates that Gal-13 exists mostly as a homodimer (Fig. 1F, Fig. S3) as determined by comparing the gel migration of Gal-1 (dimer) and Gal-13 C136S/C138S (monomer). Furthermore, an SDS-PAGE also clearly showed that dimerization (approximately 38 kDa) occurs under non-reducing conditions (Fig. 1G, Fig. S4). However, two bands (approximately 32 kDa and 28 kDa) appeared at the lower positions of the dimer band. Such behavior of Gal-13 in the SDS-PAGE has been observed before^12^ and the cause is unknown.

### The carbohydrate binding site of Gal-13

**Figure 2:**
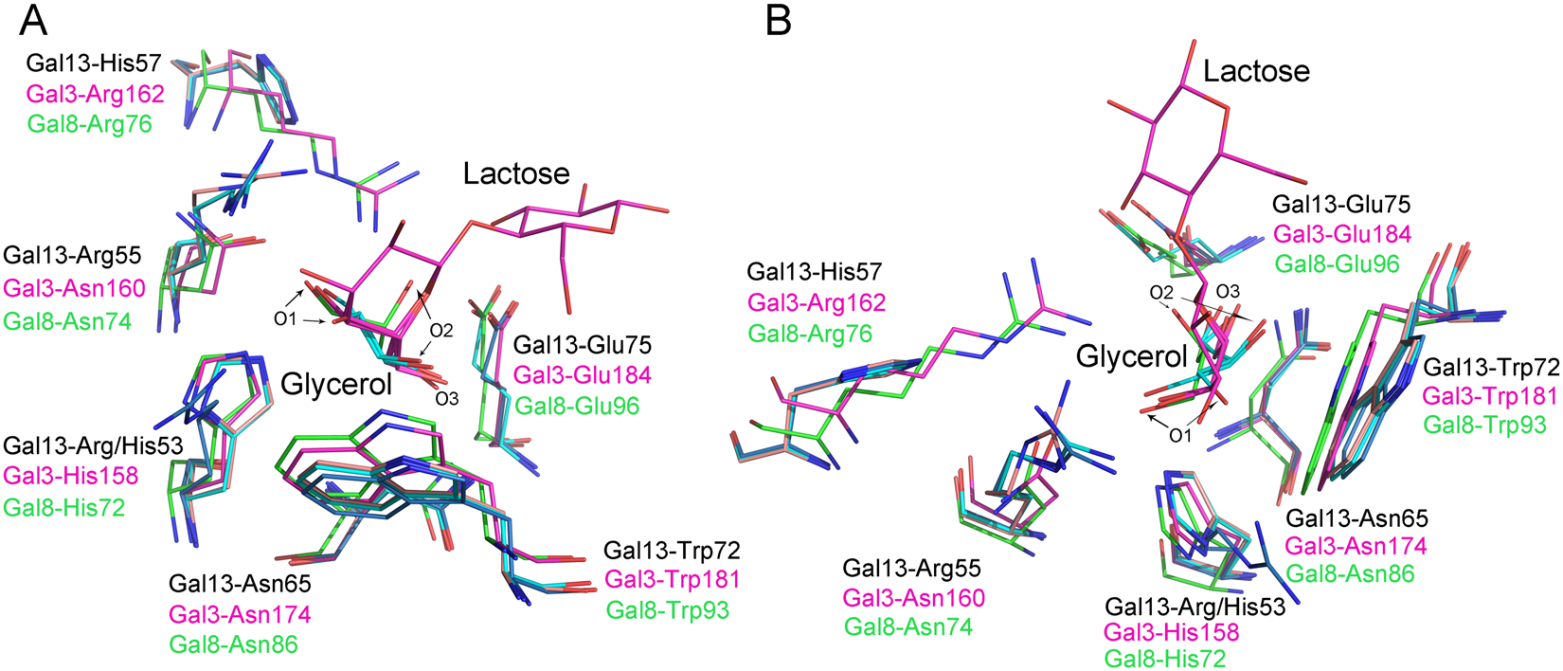
The overlay of carbohydrate binding sites for wild-type Gal-13 and its R53H mutant with the Gal-3 CRD (PDB: 2NMO, solved by Collins *et al*.^32^) and Gal-8 N-terminal CRD (PDB: 5GZC, solved by Si *et al*.^31^). *wt* Gal-13 is colored deep blue; R53H is colored cyan and wheat; Gal-3 CRD (with glycerol and lactose bound in partial occupancies) is colored purple; Gal-8 N-terminal CRD is colored green. (**A**) Side view of the overlay. Arg53 is positioned at the bottom of the *wt* Gal-13 carbohydrate binding site. A histidine is normally located at the same position in the Gal-3 CRD and the Gal-8 N-terminal CRD. Moreover, there is an arginine residue (Arg162 of Gal-3 and Arg76 of Gal-8) at the same position as His57 in Gal-13. Glycerol molecules in the carbohydrate binding sites of Gal-3 CRD and Gal-8 N-terminal CRD could exactly merge with a part of lactose. Two glycerol molecules in the carbohydrate binding site of the R53H mutant could not merge with lactose. The O1 atoms of these two glycerol molecules in the R53H carbohydrate binding site point in different directions. (**B**) Top view of the overlay.

In Gal-13, β-sheet strands S4, S5 and S6 form a concave shape that is quite similar to the carbohydrate binding sites of other galectins. However, the amino acid composition of Gal-13 in this region is somewhat different (Fig. 2). There is an arginine (Arg53) located in the middle of strand S4 (Fig. 2). In other galectins, histidine occupies this position and stabilizes interactions with the β-galactoside by forming a hydrogen bond with O4 of the sugar ligand^28^. In Gal-13, there is a histidine at position 57 (Fig. 2), whereas in Gal-3, an arginine (Arg162) is at the analogous position and forms a hydrogen bond with O3 of the galactoside residue^29^. Even with 50 mM lactose in the crystallization buffer, our Gal-13 structure lacks any electron density that is expected for a bound lactose molecule and only shows water molecules in the ligand binding site (Fig. S5). This is likely due to differences in the amino acid residues at positions 53 and 57 in Gal-13 compared to other galectins such as Gal-3 (Fig. S6). To test this hypothesis, we mutated Arg53 in Gal-13 to a histidine to assess whether we could restore lactose binding. Superposition of the crystal structures for wild type Gal-13 and the R53H mutant, along with those of Gal-3 and Gal-8, demonstrates that His53 in R53H indeed has a similar orientation as the analogous histidines in Gal-3 and Gal-8 (Fig. 2). However, we did not find lactose bound in the Gal-13 R53H mutant, even when the crystallization buffer contains 50 mM lactose. Nevertheless, the R53H variant does trap a molecule of glycerol at that site, and that trapped glycerol molecule is not observed when we flash cool a crystal of the R53H mutant in the absence of the glycerol cryoprotectant (Fig. 2). The crystallization solution contained 20% (w/v) PEG3350, which is also a good cryoprotectant. Therefore, there are no ice rings in the data collection frames. In the carbohydrate binding site of this R53H structure without any cryoprotectant, water molecules were identified (Fig. S5).

Glycerol is a flexible molecule^30^ that can adopt two different conformations within the carbohydrate binding site (Fig. 2, Fig. S7). Moreover, the C2, C3, O2, and O3 atoms of these two glycerol conformations occupy the same positions, whereas the C1 and O1 atoms are oriented differently. In one instance, the C1-O1 bond is oriented towards the conserved tryptophan (Trp72), and in the other, this bond is oriented towards Arg55. Previous structural studies of Gal-3 and Gal-8 showed that glycerol could perfectly occupy the C4, C5, C6, O4, O5 and O6 positions of the galactoside (Fig. 2)^31–33^. In this regard, the conformations of the two glycerol molecules in the R53H variant are different from those in Gal-3 and Gal-8 (Fig. 2).

### Hemagglutination Activity

**Figure 3:**
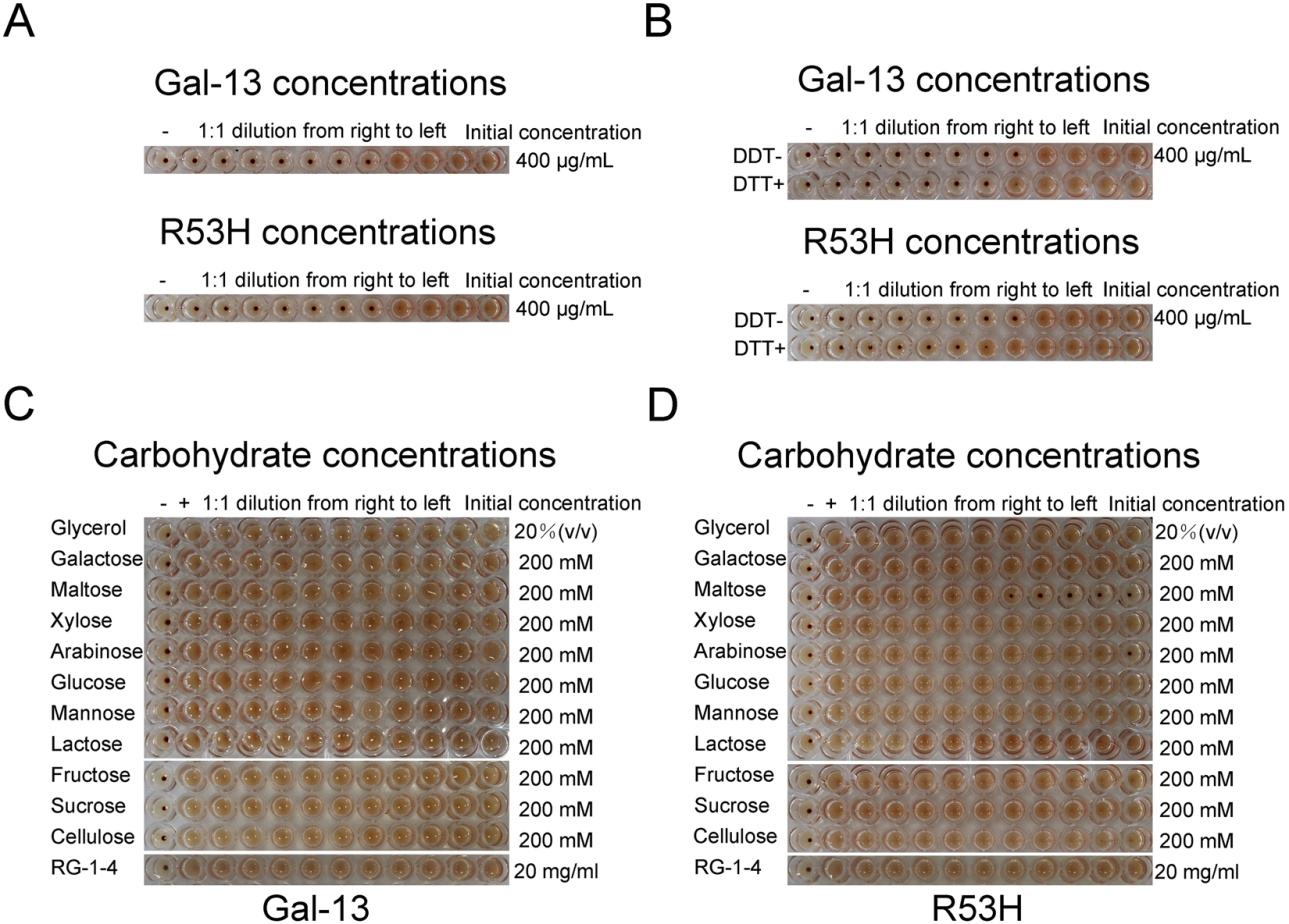
Hemagglutination Assay. (**A**) Gal-13 and R53H induce agglutination of chicken erythrocytes with an MAC (minimum agglutination concentration) value of 50 µg/ml. Panel A in this figure shows two rows of 12 wells each, the top row for Gal-13 and the bottom row for R53H. In each row, the well at the far right contains the maximum concentration used, i.e., 400 μl/ml. Moving then to the left in each row, serial dilutions were made as follows: 400, 200, 100, 50, 25, 12.5, 6.25, 3.13, 1.6, 0.8, and 0.4 μl/ml. The final well at the far left contains no protein as a negative control. Whereas 50 µg/ml Gal-13 or R53H induced agglutination, 25 µg/ml did not. Therefore, the MAC (minimum agglutination concentration) value is 50 µg/mL. (**B**) DTT did not significantly influence Gal-13-induced hemagglutination. (**C**) No carbohydrates tested could inhibit Gal-13-induced hemagglutination. (**D**) Aside from maltose and arabinose, all other carbohydrates could not inhibit R53H-induced hemagglutination.

The bio-activities of Gal-13 and R53H were assessed in the hemagglutination assay. We found that both proteins could promote chicken erythrocyte agglutination with a minimum agglutination concentration (MAC) of 50 µg/mL (Fig. 3A), consistent with a previous report^12^. We also determined that the presence of DTT did not significantly influence Gal-13-induced hemagglutination (Fig. 3B), a finding that runs contrary to that report^12^.

The inhibitory effects of galactose, maltose, xylose, arabinose, glucose, mannose, lactose, fructose, sucrose, cellulose and RG-I-4 (a polysaccharide isolated from ginseng) on Gal-13 and R53H were also determined. Whereas all carbohydrates tested failed to inhibit wild-type Gal-13-induced hemagglutination (Fig. 3C), maltose and arabinose could inhibit the biological activity of R53H with Minimum Inhibition Concentration (MIC) values of 12.5 mM and 200 mM, respectively (Fig. 3D). Because the R53H mutant could bind glycerol, we investigated whether glycerol could inhibit Gal-13- and R53H-induced agglutination and found that it could not. This result is consistent with previous reports on Gal-3 and Gal-8^31,33^ and indicates that glycerol is not a physiological ligand.

### Sugar binding tests of Gal-13

**Figure 4:**
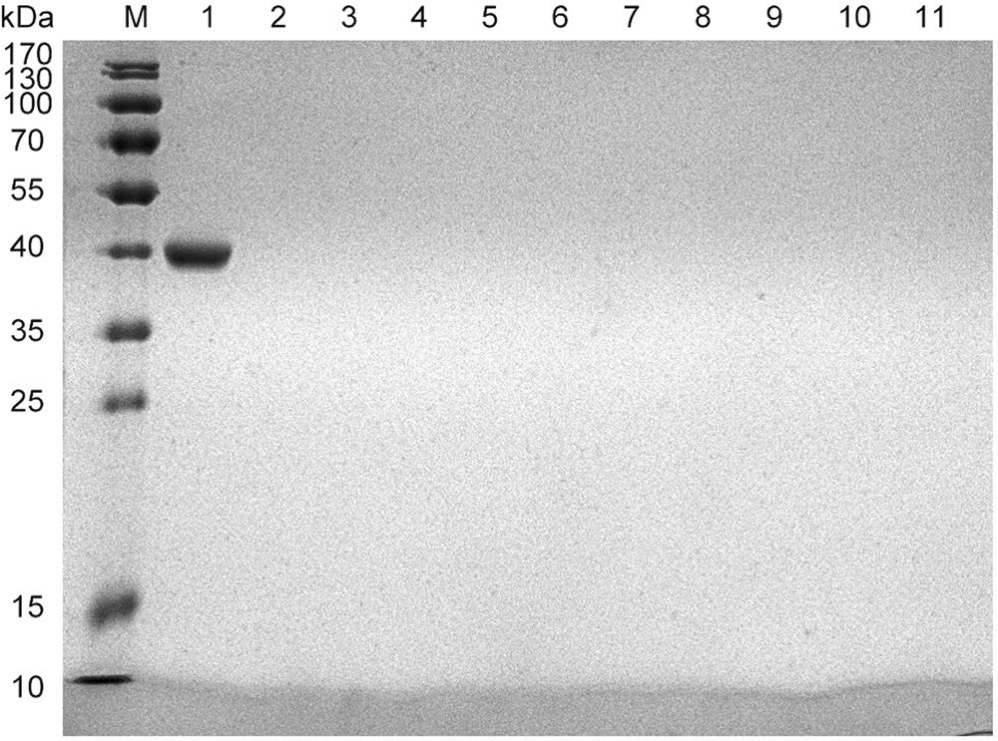
Affinity chromatography. “M” indicates a protein molecular weight marker. Lane 1 shows that lactose-modified Sepharose 6b could recover GST-Gal3_111-250 (41.5 kDa)^34^. Lane 2 shows that Sepharose 6b without modification could not recover Gal-13. Lane 3–6 indicates that lactose-, arabinose-, maltose-, and sucrose-modified Sepharose 6b also could not recover Gal-13. Lane 7 shows that Sepharose 6b without any modification could not recover R53H. Lane 8–11 indicates that lactose-, arabinose-, maltose-, and sucrose-modified Sepharose 6b also could not recover R53H.

To determine whether Gal-13 and its R53H mutant could directly bind sugars, we performed a series of affinity chromatography studies (Fig. 4). Gal-13 and R53H were assessed for their ability to bind lactose, arabinose, maltose, and sucrose in the solid phase. Experiments were performed using unmodified Sepharose 6b beads and beads modified with lactose, arabinose, maltose, and sucrose. The same sugars were used to displace any Gal-13 bound to the columns. Whereas in the control, a significant amount of GST-Gal3_111-250 (41.5 kDa) could bind to lactose-modified Sepharose 6b, SDS-PAGE showed that neither wild type Gal-13 nor its R53H variant was recovered from any of the sugar-modified columns. As a control for non-specific interactions, the unmodified sepharose 6b (negative control) also showed no binding to either Gal-13 or R53H.

**Figure 5:**
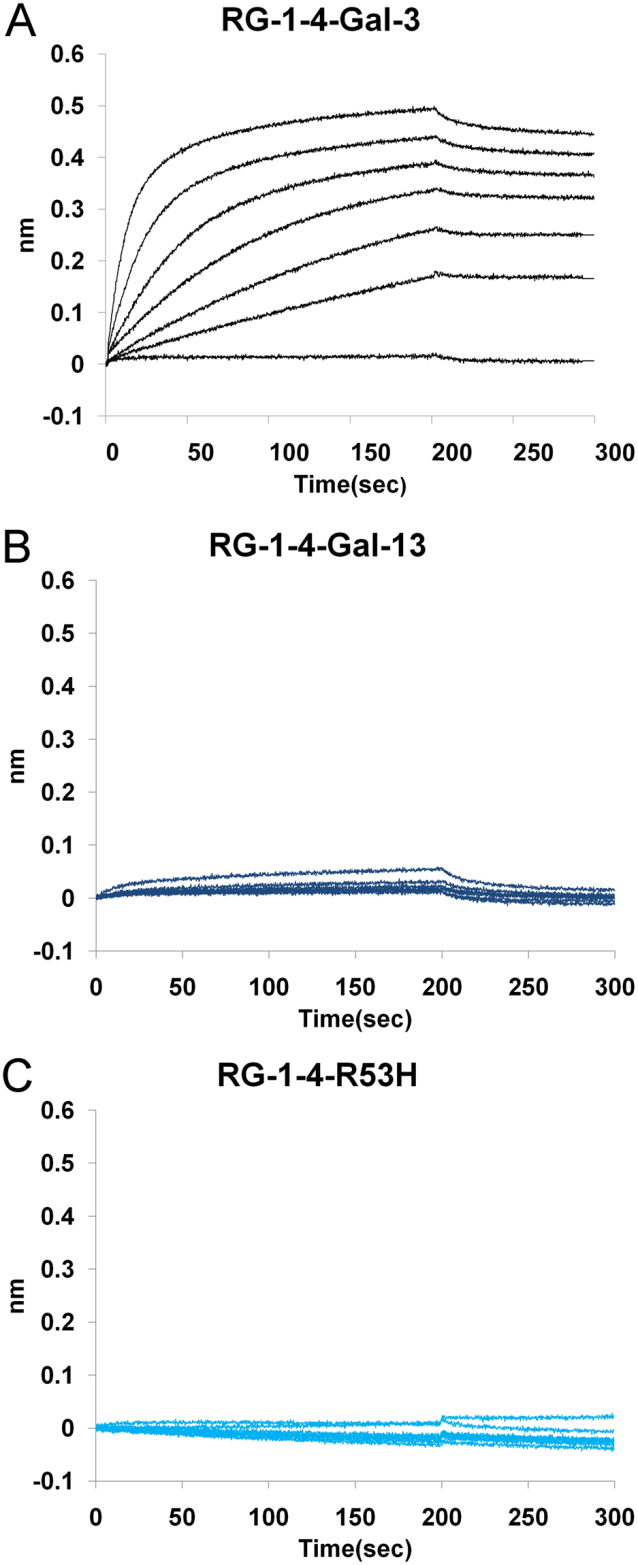
Biolayer interferometry. (**A**) The affinity of Gal-3 for RG-I-4 was determined by biolayer interferometry. The K_D_ value of Gal-3 for RG-I-4 was calculated using 1:1 binding stoichiometry to be 68 nM. (**B**,**C**) Direct interactions between Gal-13/R53H and RG-I-4 were determined by biolayer interferometry. RG-I-4 could not effectively bind to Gal-13 or R53H.

We used biolayer interferometry to determine binding affinities of Gal-13 to polysaccharides, including ginseng pectin RG-I-4 (a type of rhamnogalacturonan I pectin)^34,35^. The dissociation constant (K_D_) was determined by varying the polysaccharide concentration. Using the Ni-NTA sensor, His-tagged Gal-3 (10 μg/ml) and His-tagged Gal-13 were captured on the sensor, and binding curves for RG-I-4 are shown in Fig. 5. RG-I-4 bound strongly to Gal-3 (Fig. 5A) with a K_D_ value calculated using a 1:1 binding stoichiometry to be 68 nM. However, RG-I-4 could not effectively bind to Gal-13 or its R53H mutant (Fig. 5B,C).

### Distribution of Gal-13 in the cell

**Figure 6:**
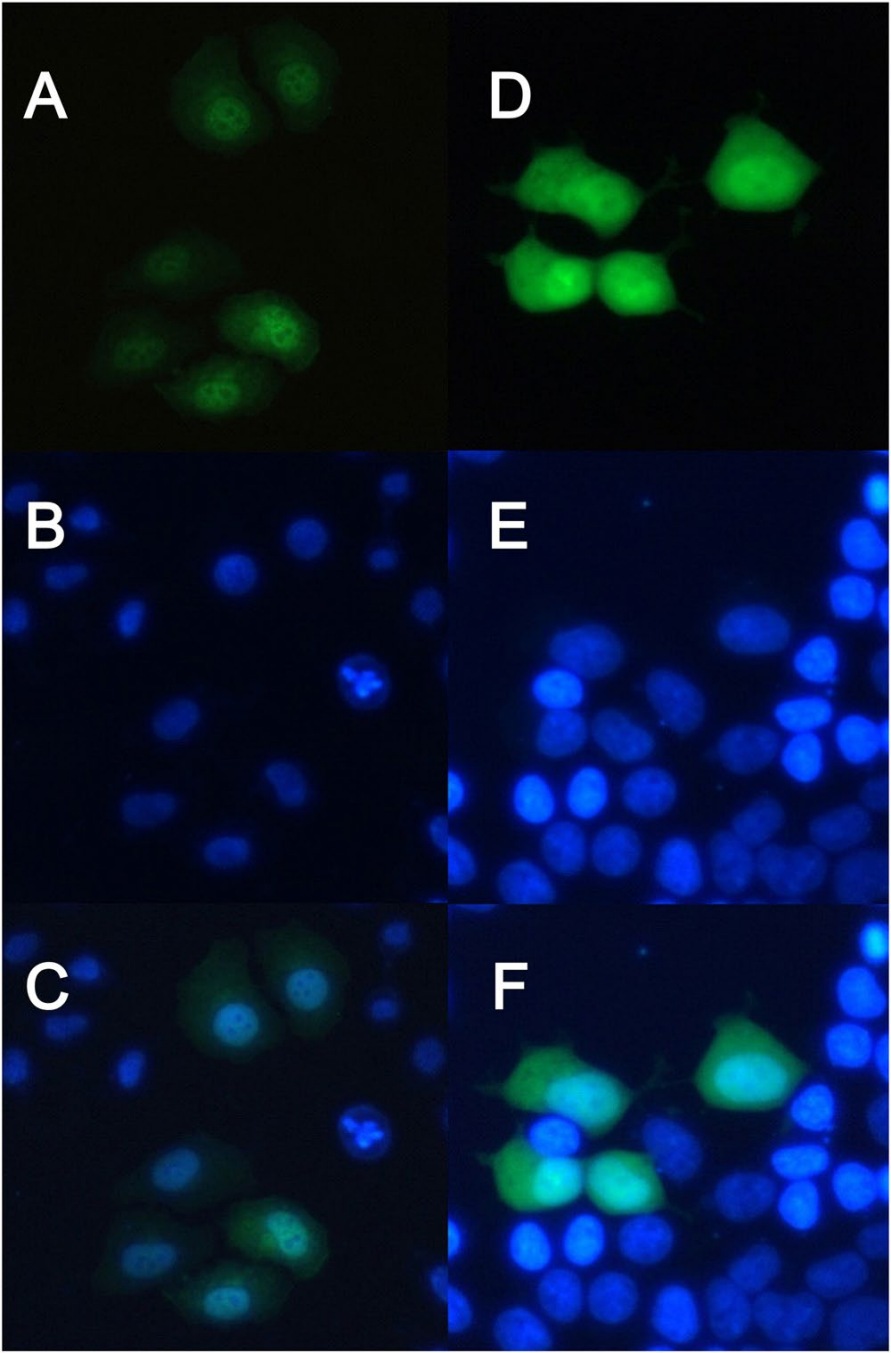
Distribution of GFP-tagged Gal-13 in HeLa and 293 T cells. (**A**) Following transfection, GFP-tagged Gal-13 was found to be distributed in the cytoplasm and nucleus. This protein was mainly localized within the nucleus of HeLa cells. There are 4–6 dark areas in the nucleus. (**B**) The nucleus of HeLa cells was stained by Hoechst. (**C**) Merge of (**A**) and (**B**). Similar experiments were performed using 293 T cells. (**D**) The GFP-tagged Gal-13 in 293 T cells is visualized. (**E**) The 293 T cell nuclei were stained using Hoechst. (**F**) Merge of (**D**) and (**E**).

To study the distribution of Gal-13 in cells, we generated an EGFP-tagged Gal-13. Based on the importance of the C-terminus to the Gal-13 dimer structure, we placed EGFP at the N-terminus of the lectin. The vector was then transfected into HeLa and 293 T cells using the PEI method, and after 16 h of culture, expression of the fusion protein was assessed by using fluorescence microscopy. Figure 6 shows that Gal-13 can exist in both the cytoplasm and the nuclear matrix of HeLa and 293 T cells. However, luminescence in the nuclear matrix was much brighter than that in the cytoplasm (Fig. 6) and merged perfectly with Hoechst staining. The strong fluorescence signals from Gal-13 in the nuclear matrix implies that the lectin likely plays some role in regulating gene expression. Figure 6 also shows that each nucleus contains 4 or 6 nucleoli that exhibit much lower fluorescence signals than the nuclear matrix.

## Discussion

Here, we report the crystal structures of Gal-13 and its R53H variant and assess their ligand binding specificities. We found that Gal-13 forms a homodimer structure that is stabilized primarily by the presence of two disulfide bonds, unlike the structures of other prototype galectins. Although this finding is consistent with a previous report that Gal-13 can form dimers via disulfide bond formation^12^, our study revealed that it is the inter-subunit interactions between Cys136 and Cys138 that allow the two disulfide bridges to form. In addition, we found that C-terminal residues Val135, Val137, and Asn139 contribute six hydrogen bonds to stabilize the Gal-13 dimer. Interestingly, a recently identified natural variant of Gal-13 that lacks the entire F1 strand (delT221), cannot dimerize, and women that express this variant are prone to severe pre-eclampsia^13,27^, which implies that Gal-13 dimers play a critical role in gestation.

**Figure 7:**
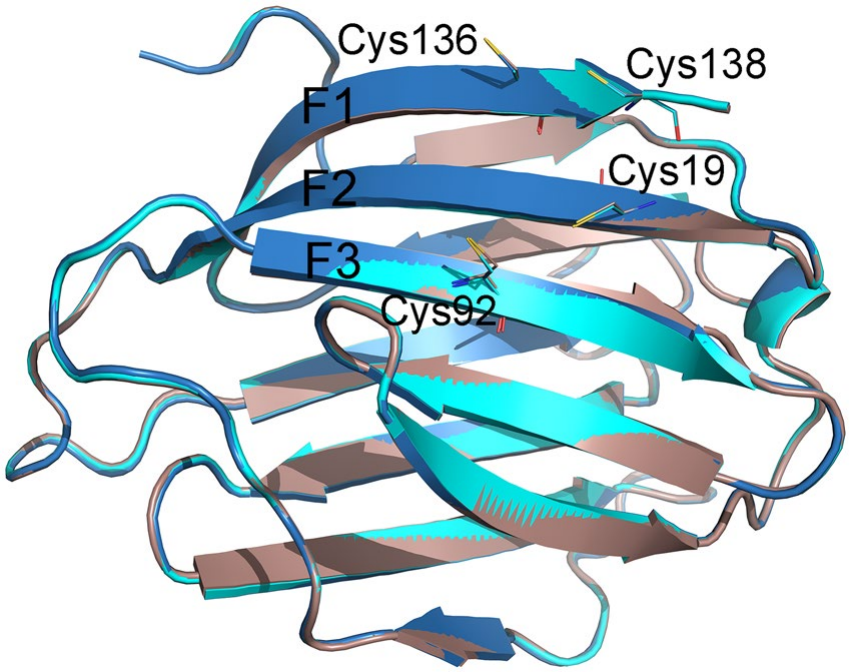
The positions of Cys19, Cys92, Cys136, and Cys138. *WT* Gal-13 is colored deep blue; the two R53H structures are colored cyan and wheat.

Galectin-1 also forms a dimer that involves the F1 strand, as well as the S1 strand, and has cysteine residues in both of these strands. Native PAGE and gel filtration results demonstrate that Gal-13 can exist in both dimer and monomer states, whereas Gal-1 normally exists as a dimer at micromolar concentrations^36^. However, Gal-1 has two cysteines located at its termini (Cys2 and Cys130, Fig. S6), both of which remain in a reduced state and neither of which plays a role in dimerization as in Gal-13. Nevertheless, a mutant of Gal-1 (C2S) has been reported to exist primarily as a monomer with a lower ligand binding affinity compared to wild-type Gal-1^37^. Cys2 in Gal-1 is in the S1 β-strand at the dimer interface, and the crystal structure of Gal-1 C2S shows that this mutation induces a global change in the Gal-1 structure that impacts its ability to bind carbohydrates^38^. Aside from Cys2, Gal-1 has five other cysteines (Cys16, Cys42, Cys60, Cys88, and Cys130). Cys130 is located in the F1 strand^38^, in a similar position to that of Cys136 in Gal-13. The distance between the sulfur atoms of the two Cys130 residues in the two monomer subunits of the Gal-1 dimer is approximately 4.3 Å^38^. This distance would not allow the two Cys130 residues to form a disulfide bond, and Gal-1 dimers can therefore dissociate into monomers at relatively low concentration^36^. In addition, although the other cysteines in Gal-1 can form disulfide bonds (Fig. 7) in the absence of reducing agent, doing so leads to a loss of Gal-1 activity^39–41^.

In addition to Cys136 and Cys138, Gal-13 has two other cysteine residues Cys19 and Cys92, whose positions in the sequence are conserved in Gal-1 with Cys16 and Cys88 (Fig. S6). Even though the distance between these cysteines would allow formation of intra-molecular disulfide bonds, this is not observed. Moreover, Cys19 is proximal to both Cys136 and Cys138 and could influence or inhibit Gal-13 dimerization by promoting different disulfide bond formation, yet it does not (Fig. 7). Two high-resolution co-crystal structures of a peptide and the Gal-8 C-terminal CRD show that residues on the F-face interact directly with the peptide^42,43^. Moreover, the F face of Gal-1 and Gal-3 CRD can bind to saccharides^44,45^. Therefore, one should not discard the possibility that the Gal-13 CRD F-face is involved in some type of ligand interaction.

In general, prototype galectins, other than Gal-13, dimerize via non-covalent interactions. Gal-1 and Gal-2 have similar quaternary structures with their dimers being stabilized by hydrogen bonds and electrostatic interactions between β-strands F1 and S1^38,46^. The Gal-7 dimer is different in that its dimer interface is formed by association of the F-faces of two CRDs, an interaction that is stabilized by hydrogen bonds and van der Waals interactions^47^. Although Gal-10 crystallizes as an apparent monomer^48–50^, careful inspection of crystal contacts suggests that it may also form a dimer. Overall, the different quaternary structures of prototype galectins suggest that they may have different ligand binding specificities *in vivo*.

Residues at the carbohydrate binding site of Gal-13 are different from those of other galectins. Alignment of residues from all other galectins shows that a histidine is crucial for binding lactose, yet in Gal-13 this position is substituted by an arginine (i.e., Arg53) (Fig. 2, Fig. S6). Our Gal-13 structure shows that Arg53 protrudes from the base of the carbohydrate binding site, and the characteristics of an arginine, including its size and charge, are obviously different from those of a histidine. This difference suggests that wild type Gal-13 should not be able to bind galactose, which likely explains why we could not find lactose bound to Gal-13 in crystals exposed even to 50 mM lactose. However, this does not exclude the possibility that Gal-13 can bind monosaccharides or even amino acid residues. In an attempt to restore β-galactoside ligand binding of Gal-13, we made the R53H mutant, and its structure showed that a molecule of glycerol binds at that site. However, the orientation and conformation of glycerol in Gal-13 are also different from those in the carbohydrate binding sites of Gal-3 and Gal-8 N-CRD^31,32^.

Similar to other galectins, Gal-13 can induce erythrocyte agglutination^12^. However, our research shows that galactose, maltose, xylose, arabinose, glucose, mannose, lactose, fructose, sucrose, and cellulose do not inhibit Gal-13-mediated agglutination. There are three explanations for this result: 1) Gal-13 induces erythrocyte agglutination via protein-protein interactions; 2) Gal-13 interacts so strongly with glycans on the erythrocyte membrane that monosaccharides/disaccharides cannot inhibit them; or 3) that Gal-13 interacts with cell surface glycans in a non-classical fashion, e.g., by binding through their F-faces^44,45^. Regardless, this phenomenon is not unique. In our studies on Gal-2, high concentrations of β-galactosides also could not inhibit Gal-2-induced hemagglutination^46^. Our Gal-13 crystal structure shows that dimers are stabilized primarily by formation of two disulfide bonds. However, the presence of DTT could not inhibit Gal-13- or R53H-induced erythrocyte agglutination, a finding that runs contrary to a previous report^12^. This conundrum may be explained by our gel filtration studies that demonstrate that Gal-13 can exist as a dimer even in the presence of DTT and thus promote agglutination by glycan cross-linking. Interestingly, 12.5 mM maltose and 200 mM arabinose can effectively inhibit R53H-mediated bio-activity. In this regard, the crystal structure of the Gal-13 R53H mutant indicates that glycerol is trapped in the binding pocket, implying that it may be sensitive to carbohydrates such as maltose and arabinose. However, we found that glycerol has no effect on R53H-induced erythrocyte agglutination. Other reports also demonstrated that glycerol cannot inhibit Gal-3^32^ or Gal-8 N-CRD^31^ induced hemagglutination, even though glycerol can co-crystallize in the carbohydrate binding sites of both lectins^31,32^

Our finding that Gal-13 could not bind to Sepharose 6b covalently linked with lactose, arabinose, maltose, or sucrose seems inconsistent with reports that Gal-13 can bind to sugar-modified agarose gels (N-acetyl-lactosamine, mannose, N-acetyl-galactosamine, maltose, glucose, fucose, and lactose-coupled agarose beads)^12^. Nevertheless, Gal-10 with 54% sequence homology to Gal-13, also could not bind strongly to saccharide-modified Sepharose^49^. Our BLI results indicate that Gal-3 can bind to the RG-I-4 polysaccharide, and RG-I-4, with its rich galactan domain, shows high affinity for the Gal-3 CRD^34,51^. However, Gal-13 also can not bind to this polysaccharide. In this regard, our results indicate that Gal-13 may be unique to the galectin family.

In conclusion, the crystal structure of Gal-13, a new prototype galectin, has been solved. We report here that Gal-13 forms a new type of dimer structure that is different from that found with other prototype galectins. Moreover, our biochemical results indicate that Gal-13 may not bind carbohydrates, a proposal that is consistent with previous work demonstrating that Gal-13 purified from human term placenta contains no carbohydrate at all^10^. Because Gal-13 expression is related to abnormalities in pregnancy, it is crucial to identify its natural ligand *in vivo*. Overall, our Gal-13 structures and ligand binding findings provide some clues and suggestions for future studies.

## Methods

### Ethics statement

All animal experiments were performed in agreement with the recommendations in the Guide for the Care and Use of Laboratory Animals of China Association for Laboratory Animal Science. The Ethics Committee of Northeast Normal University approved all animal care and handling protocols (Changchun, China).

### Cloning, Expression, and Purification of Gal-13

The gene for Gal-13 (residues 1–139, uniprot code: Q9UHV8) was synthesized by SynBio Technologies (Monmouth Junction, NJ, USA). Gal-13 was amplified using the following primers: Gal-13 forward: 5′- GGATCCCATATGAGCAGTCTGCCGGTT - 3′, Gal-13 reverse: 5′- GGATCCCTCGAGTTAATTGCAGACGCAAACGCT- 3′, which contain *Nde*I and *Xho*I restriction sites, respectively. PCR products were digested with *Nde*I and *Xho*I and cloned into a pET28a vector (Novagen, Gibbstown, NJ, USA). Site-directed mutagenesis of Gal-13 was carried out with the QuickChange XL site-directed mutagenesis kit (Stratagene). A His-tag was constructed in the N-terminus of Gal-13. The recombinant plasmid was confirmed by DNA sequencing. *Escherichia coli* BL21 (DE3) cells were transformed with this recombinant plasmid and induced to express proteins by incubating with 0.5 mM IPTG for 16 h at 25 °C. The protein was extracted and purified using a Ni-NTA agarose column (Qiagen, Hilden, Germany) according to previously reported protocols^46^. Following purification, the Gal-13 protein was dialyzed in 10 mM HEPES, pH 7.0. During overnight dialysis at 4 °C, thrombin was added to remove His tags from the Gal-13 protein. Each milligram of His-tagged protein was digested with 20 units (NIH unit) of thrombin. As determined by sodium dodecyl sulfate-polyacrylamide gel electrophoresis (SDS-PAGE), the purity of the resulting protein was >90%. Finally, proteins were concentrated to 5 mg/mL using an Amicon Ultra-15 Centrifugal Filter Unit (3 kDa cut off) and stored at −80 °C. R53H and C136S/C138S were generated by a site-directed mutagenesis kit (New England Biolabs). The described procedures were the same as those used for the wild type lectin.

### Crystallization, Data Collection, and Structure Determination

Hampton Research (Aliso Viejo, CA, USA) packs (PEGRx1, PEGRx2, Index, Crystal Screen, and Crystal Screen 2, SaltRx, SaltRx2, PEG/ Lon screen, PEG/ Lon 2 screen, matrix 1/ 2) were used for the initial crystallization screen (sitting-drop vapor diffusion method). To obtain a crystal that was suitable for X-ray diffraction, we used the hanging-drop method. Gal 13 crystals were obtained between 7 and 14 days from drops that contained 1 µl protein and 1 µl solution from the well containing 0.1 M Tris-HCl, pH 8.5, 0.05 M lactose, 0.2 M MgCl_2_, 30% (w/v) PEG 4000 at room temperature. R53H crystals were obtained after 14 days from drops that contained 1 µl protein and 1 µl solution from the well containing 0.1 M Tris-HCl, pH 8.5, 0.05 M lactose, 0.2 M NaF, 20% (w/v) PEG 3350 at room temperature. Prior to X-ray data collection, Gal-13 and R53H crystals were soaked for approximately 1 min in the reservoir solution supplemented with 20% (v/v) glycerol as a cryoprotectant and then flash cooled in liquid nitrogen. Another R53H crystal was not soaked in any cryoprotectant before flash cooling. Data sets were collected at 100 K at the Shanghai Synchrotron Radiation Facility 18U1 (Shanghai, China).

Data sets were indexed and integrated using XDS^52^ and scaled using Aimless^53^ from the CCP4 package (6.4.0)^54^. Structures were determined by Phaser^55^ with a molecular replacement method using the structure of Gal-10 (PDB: 1LCL)^49^ as the search model. Structure refinement and water updating were performed using Phenix^56^ refine and manual adjustment. Final structure validations were performed using MolProbity^57,58^.

### Native PAGE

The 5 ml 15% separating gel contains 1.15 ml H_2_O, 2.5 ml of a 30% (*v/v*) mixture of acrylamide and bis-acrylamide, 1.3 ml 1.5 M Tris-HCl, pH 8.8, 0.05 ml 10% (*m/v*) ammonium peroxodisulfate, 0.002 ml TEMED. The 2 ml stacking gel contains 0.69 ml H_2_O, 0.17 ml of a 30% (*v/v*) the mixture of acrylamide and bis-acrylamide, 0.13 ml 1 M Tris-HCl, pH 6.8, 0.01 ml 10% (*m/v*) ammonium peroxodisulfate, 0.001 ml TEMED. A total of 1 L electrophoresis buffer contains 14.4 g glycine and 3.02 g Tris. Proteins were mixed with 4 × native PAGE loading buffer containing bromophenol blue (0.1%, w/v), 0.75 M Tris-HCl, pH 6.8) and glycerol (30%, v/v). To each lane of a gel, 5 μg of protein was loaded. The electrophoresis ran at 90 V for 3 h.

### Gel filtration

Gel filtration was performed at 25 °C using an ÄKTA purifier 10 system (GE Healthcare, Uppsala, Sweden), with a running buffer of PBS (137 mM NaCl, 2.7 mM KCl, 10 mM Na_2_HPO_4_, 10 mM KH_2_PO_4_, pH 7.4). A total of 100 μg of protein sample was loaded onto a Superdex 75, 10/300 column (The bed volume is approximately 24 ml) and was eluted at a flow rate of 0.5 ml/min. The absorbance was monitored at 280 nm. Five standards proteins or compound (bovine serum albumin (67.0 kDa), ovalbumin (43.0 kDa), ribonuclease A (13.7 kDa), aprotinin (6.5 kDa) and vitamin B12 (1.4 kDa) were used to generate a standard curve for calculating Gal-1 and Gal-13 molecular weight. Thyroglobulin (669.0 kDa) was used to determine the void volume of the column.

### Hemagglutination Assay

The hemagglutination assay was performed as previously reported^31,46^ with some minor modifications. Briefly, chicken erythrocytes were prepared from fresh blood collected in Alsever’s medium (0.8% sodium citrate, 2.05% glucose, 0.42% sodium chloride, and 0.055% citric acid) and were washed five times with 0.15 M NaCl. Cells were then suspended in 4% (v/v) PBS (pH 7.4) containing 1 mg/mL trypsin and incubated at 37 °C for 1 h. After washing with 0.15 M NaCl, the cells were fixed in PBS buffer (pH 7.4) for 1 h containing 1% glutaraldehyde at room temperature followed by termination with five volumes of 0.1 M glycine in PBS buffer (pH 7.4). The fixed cells were washed and adjusted to 10% (v/v) with PBS buffer (pH 7.4). The hemagglutination assay was performed in microtiter V plates, with each well containing 75 µl Gal-13 or 75 µl control solution and 25 µl 4% (v/v) chicken erythrocyte suspensions. The cells were added last, followed by shaking, and agglutination was allowed to proceed for 1 h on the ice to ensure the temperature was consistent.

### Biolayer interferometry

The affinity of polysaccharide for Gal-13 was measured using a ForteBio Octet RED 96 instrument. Ni-NTA biosensors (Fortebio) were hydrated with PBS for 20 min prior to performing the experiment. The concentration of His-tagged Gal-13 were both 10 μg/ml, and monitoring was conducted as follows: initial baseline for 60 s, loading for 100 s, baseline for 90 s, association approximately for 150–300 s, and dissociation for 60 s. The regeneration buffer was 10 mM glycine (pH 2.0), and recharging (10 mM NiCl_2_ in ddH_2_O) was done for 60 s. To determine binding kinetics, seven concentrations of each sample were dissolved in PBS. To determine binding parameters, data were analyzed using ForteBio Data Analysis Software 7.0.2.8. The affinity of polysaccharide for Gal-3 was used as a control.

### Sugar Binding Assays

Lactose, arabinose, maltose, and sucrose-agarose beads were generated according to Nermin Fornstedt’s method^59^. Gal-13 (25 µg) was added to 50 µl lactose, arabinose, maltose, and sucrose-Sepharose 6b beads. The solution was incubated in 1.5-ml microtubes with gentle stirring at 4 °C for 2 h. Tubes were then centrifuged at 1000 × g for 1 minute to sediment the agarose beads. The beads were washed in buffer containing 135 mM NaCl, 2.7 mM KCl, 1.5 mM KH_2_PO_4_, and 10 mM Na_2_HPO_4_. Bound protein was eluted with 300 mM lactose, arabinose, maltose, and sucrose, sequentially.

### Transfection and Fluorescence microscopy

To assess the subcellular localization of Gal-13, we transiently transfected Gal-13 into 293 T and HeLa cells by using PEI. Gal-13 was cloned into a pEGFP-C2 vector. EGFP was constructed in the N-terminus of Gal-13. 293 T and HeLa cells were maintained in Dulbecco’s modified Eagle’s medium (DMEM) supplemented with 10% newborn bovine serum and 100 units/ml of penicillin-streptomycin at 37 °C in an atmosphere of 5% CO2/air. The 293 T cells and HeLa cells were plated in three wells of a 6-well plate in 2 ml complete medium at a density of 2–3 × 10^5^/mL, and the medium was completely changed before transfection. A volume of 3 μg of plasmid and 3 μg of PEI were diluted, with 300 μL of blank medium per well and mixed completely and incubated for 20 minutes at room temperature. The mixture with pEGFP-C2-Gal-13 was placed into the wells. After 6 hours of transfection, the medium was replaced with complete DMEM and continuously maintained at 37 °C in a humidified atmosphere of 5% CO_2_/air. Transfection efficiency was determined by fluorescence microscopy at 24 hours at room temperature.

### Data availability

Crystallographic data that support the findings of this study have been deposited in the Protein Data Bank with the access codes 5XG7, 5XG8 and 5Y03. All data supporting our findings are available within the article or from the corresponding author upon request.

### Accession code

The PDB codes for Gal-13 structures are 5XG7, 5XG8 and 5Y03.

## Electronic supplementary material

Supplementary information
