# Supplementary material for: Galectin-13, a different prototype galectin, does not bind β-galacto-sides and forms dimers via intermolecular disulfide bridges between Cys-136 and Cys-138
Source: Sci Rep. 2018 Jan 17;8:980. doi: 10.1038/s41598-018-19465-0 (PMC5772480; doi:10.1038/s41598-018-19465-0)
Supplement: Supplementary file 1 — Supplementary information [file 41598_2018_19465_MOESM1_ESM.pdf]

Galectin-13, a different prototype galectin, does not bind β-galactosides and forms dimers via intermolecular disulfide bridges between Cys-136 and Cys-138

Jiyong Su, Yue Wang, Yunlong Si, Jin Gao, Chenyang Song, Linlin Cui, Runjie Wu, Guihua Tai*, Yifa Zhou*

Jilin Province Key Laboratory for Chemistry and Biology of Natural Drugs in Changbai Mountain, School of Life Sciences, Northeast Normal University, Changchun 130024, PR China.

Correspondence and requests for materials should be addressed to Guihua Tai (E-mail: taigh477@nenu.edu.cn) or Yifa Zhou (E-mail: zhouyf383@nenu.edu.cn).

Su *et al.*, Fig. S1

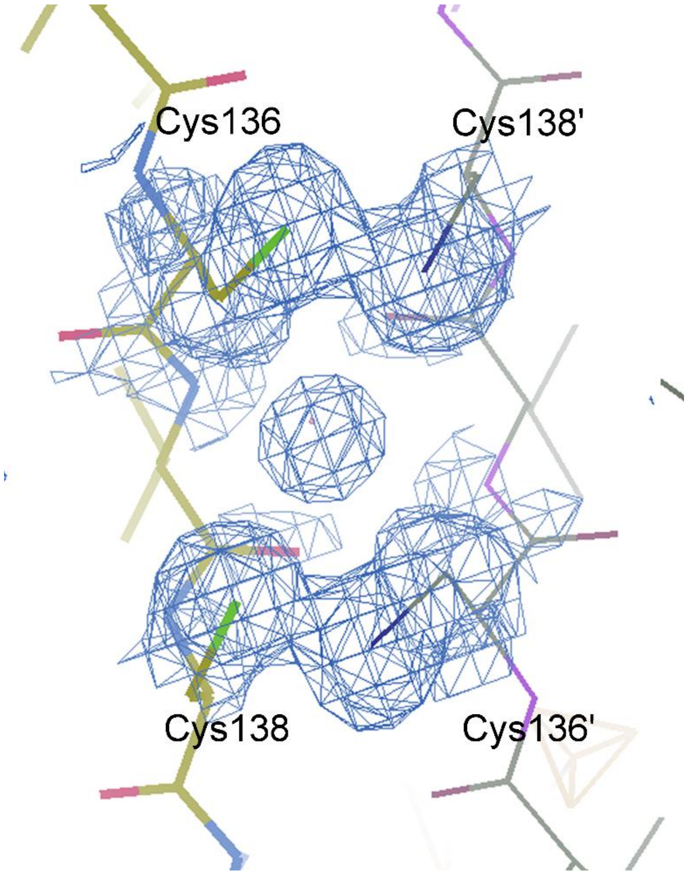

Figure S1. Electron density map of two disulfide bonds formed by Gal-13 Cys136 and Cys138. The 2|F_o|-|F_c|, αc map contoured at 1.0 δ is shown in blue. The radius of the electron density map was set to 6 Å around the cysteine residues.

Su *et al.*, Fig. S2

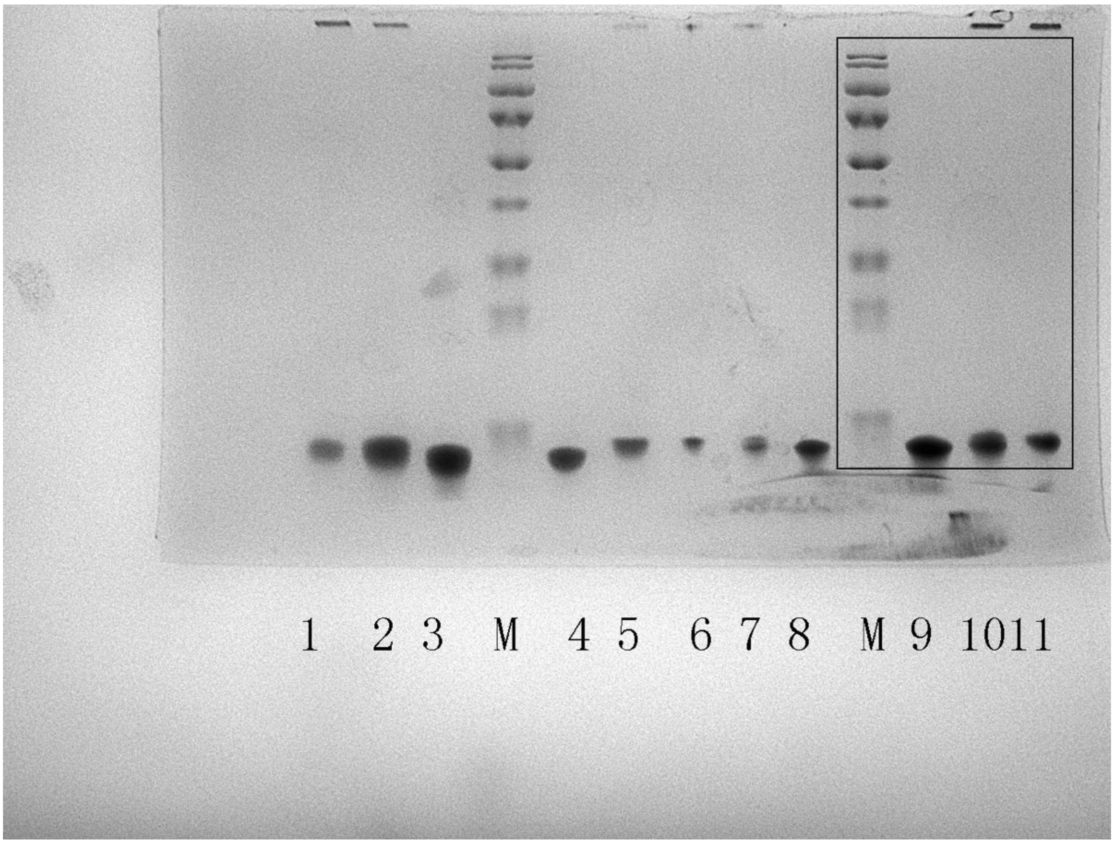

Figure S2. Normal SDS-PAGE of Gal-1, Gal-13 and the double-mutant C136S/C138S.

Fig. 1C was generated from this gel using the part marked by black lines. "M" indicates protein molecular weight markers. In lane 1, only 2 μg C136S/C138S was loaded onto SDS-PAGE, thus accounting for the low level of staining. In the other lanes, higher amounts of protein were loaded onto the gel as follows: Lane 2: 10 μg *wt* Gal-13. Lane 3: 10 μg Gal-1. Lane 4: 2.5 μg Gal-1. Lane 5: 2.5 μg *wt* Gal-13. Lane 6: 2.5 μg C136S/C138S. Lane 7: 2.5 μg *wt* Gal-13. Lane 8: 2.5 μg Gal-1. Lane 9: 5 μg Gal-1. Lane 10: 5 μg *wt* Gal-13. Lane 11: 5 μg C136S/C138S.

Su *et al.*, Fig. S3

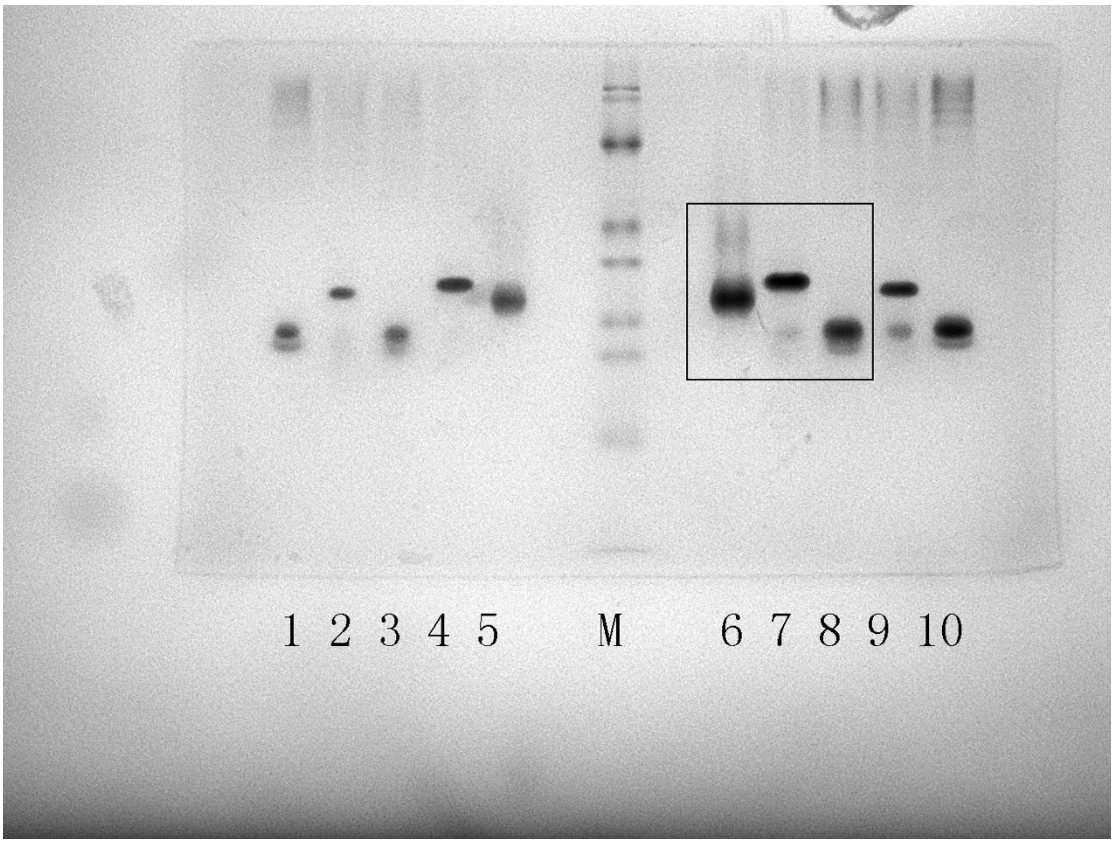

Figure S3. Native PAGE of Gal-1, Gal-13, C136S/C138S and two other proteins. Fig. 1F was generated from this gel using the part marked by black lines. Gal-1 and *wt* Gal-13 mainly form dimers (see lane 6 and 7). However, some Gal-13 monomer could also be observed. For the double mutant C136S/C138S, only monomer was observed (see lane 8). “M” indicates SDS-PAGE protein molecular weight markers. We used these markers even though they are not optimal for native PAGE. In each lane, the following proteins and concentrations were used: lane 3, 2.5 μg C136S/C138S; lane 4, 2.5 μg *wt* Gal-13; lane 5, 2.5 μg Gal-1; lane 6, 5 μg Gal-1; lane 7, 5 μg *wt* Gal-13, and lane 8, 5 μg C136S/C138S. Lanes 1, 2, 9 and 10: proteins N/A to this paper.

Su *et al.*, Fig. S4

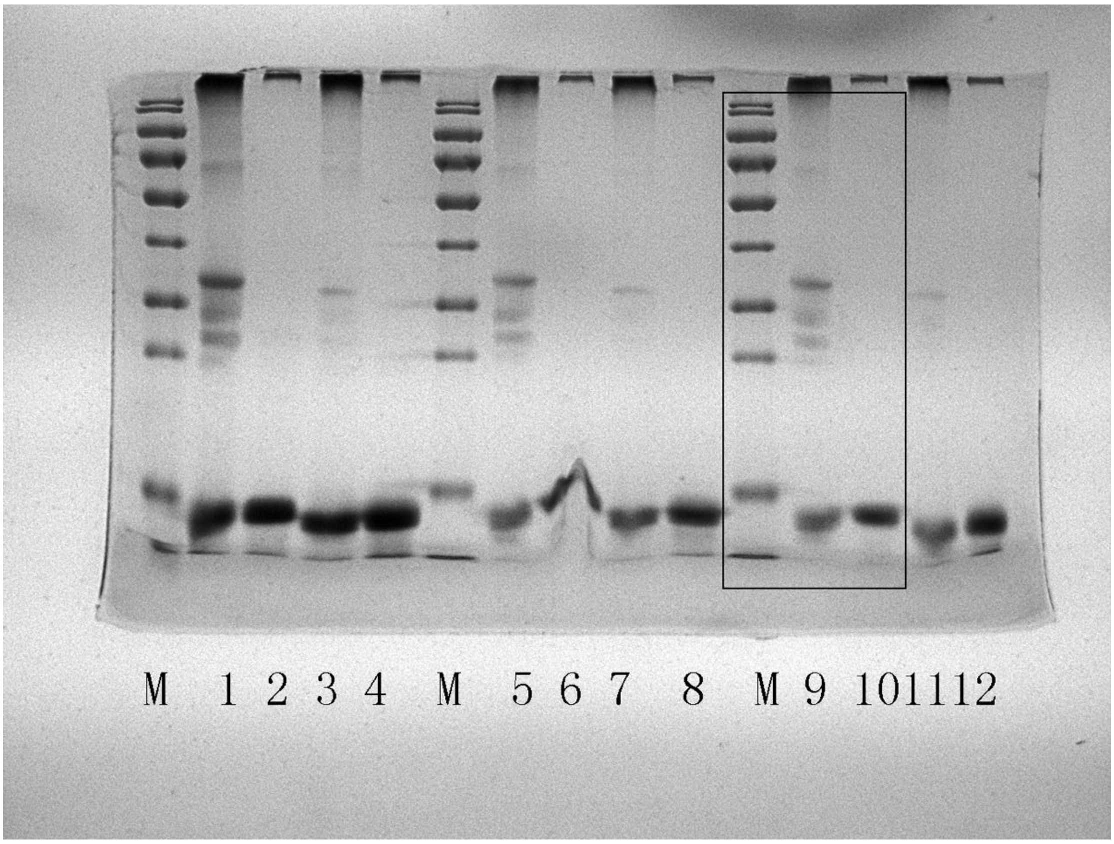

Figure S4. SDS-PAGE of Gal-13 and R53H with or without DTT reducing. Fig. 1G was generated from this gel using the part marked by black lines. M indicates protein molecular weight markers. Without DTT, Gal-13 could form a dimer (see lane 9). However, Gal-13 was reduced to monomer when DTT was added into SDS-PAGE running buffer (see lane 10). Lane 1: 10 μg *wt* Gal-13 without DTT reducing. Lane 2: Lane 1: 10 μg *wt* Gal-13 with DTT reducing. Lane 3: 10 μg R53H without DTT reducing. Lane 4: 10 μg R53H with DTT reducing. Lane 5: 7.5 μg *wt* Gal-13 without DTT reducing. Lane 6: 7.5 μg *wt* Gal-13 with DTT reducing. Lane 7: 7.5 μg R53H without DTT reducing. Lane 8: 7.5 μg R53H with DTT reducing. Lane 9: 5 μg *wt* Gal-13 without DTT reducing. Lane 10: 5

μg *wt* Gal-13 with DTT reducing. Lane 11: 5 μg R53H without DTT reducing. Lane 12: 5 μg R53H with DTT reducing.

Su *et al.*, Fig. S5

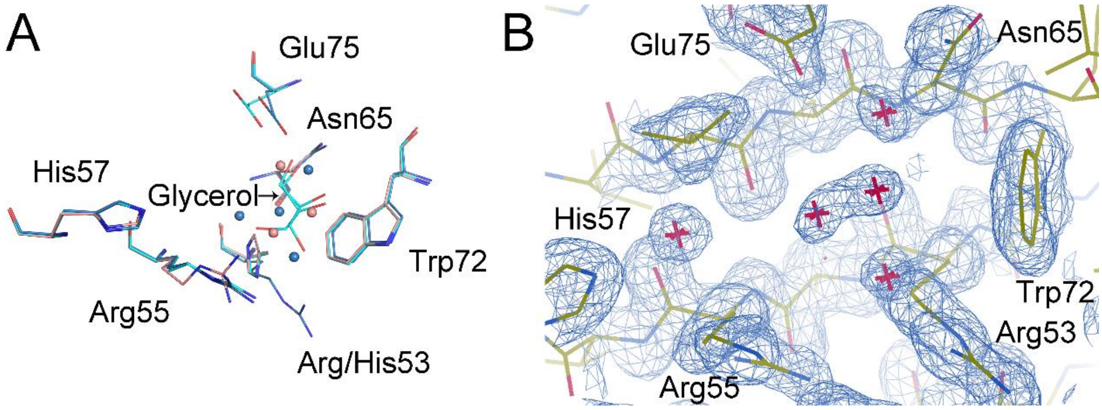

Figure S5. A. The ligand binding sites for wild-type Gal-13 and its R53H mutant are overlaid. *wt* Gal-13 is colored dark blue; R53H is colored cyan or wheat. Four blue-colored balls and three wheat-colored balls are water molecules identified in the ligand binding site of *wt* Gal-13 and R53H, respectively. **B.** The electron density map of ligand binding site for wild-type Gal-13. The $2|F_o|-|F_c|$, αc map contoured at 1.0 δ is shown in blue.

Su *et al.*, Fig. S6

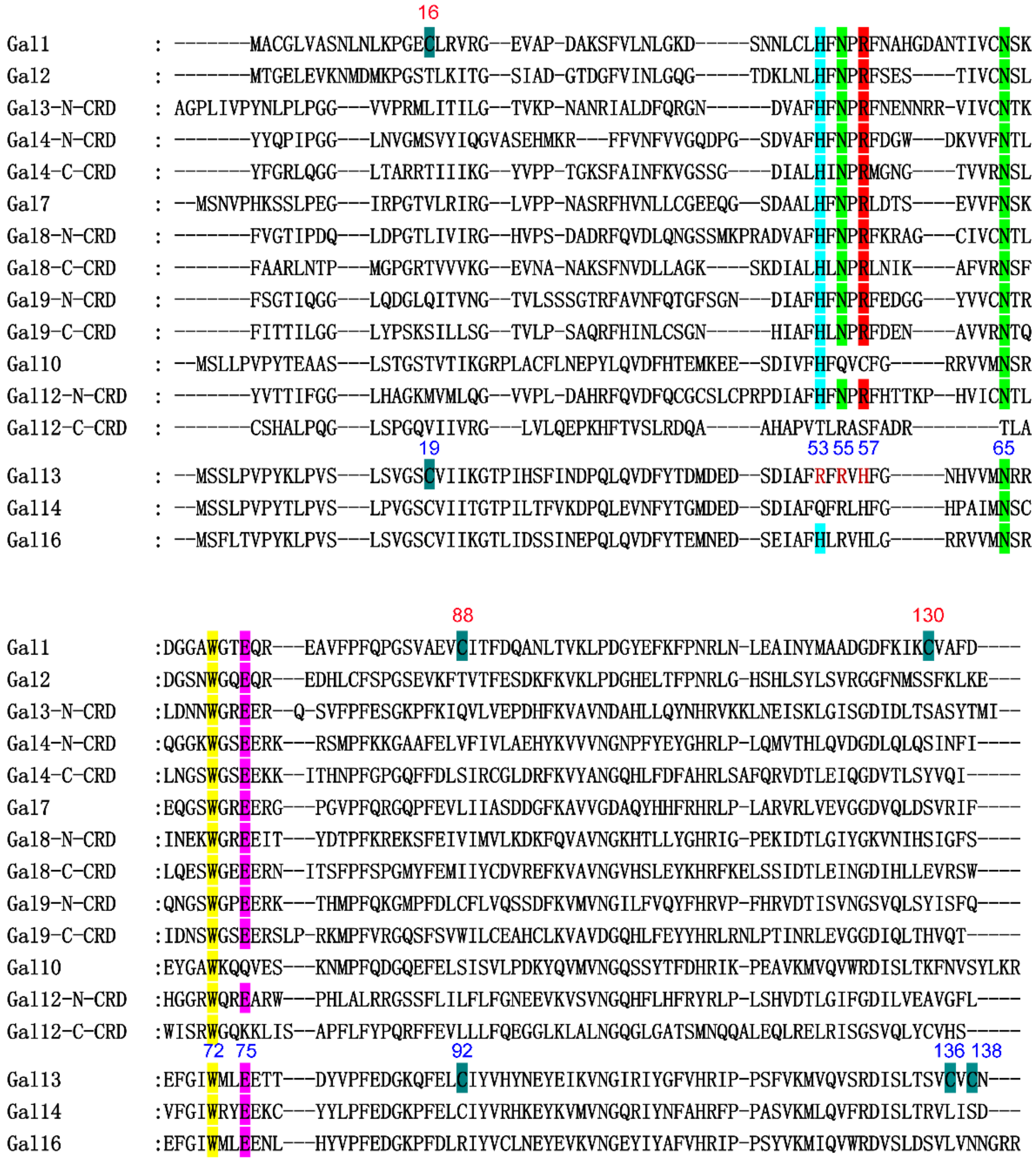

Figure S6. Alignment of the primary structures of human galectin CRDs. The alignment was generated using the program ClustalX 2.1. For Gal-13, the positions of the four cysteines and six ligand binding residues are colored blue. For Gal-1, the residues positions of three cysteines are colored red. In Gal-1, Cys130 is located at the C-terminus, and in Gal-13,

Cys136 and Cys138 are located at the C-terminus. The positions of Cys16 and Cys88 in Gal-1 are conserved in Gal-13 with Cys19 and Cys92.

Su *et al.*, Fig. S7

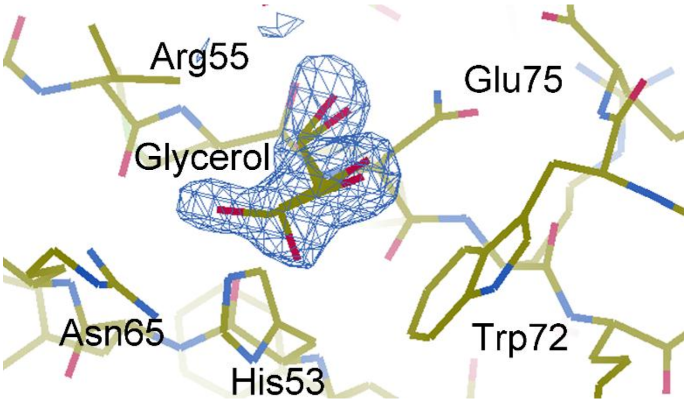

Figure S7. Electron density map showing two glycerol molecules in the ligand binding site. The 2|F_o|-|F_c|, αc map contoured at 1.0 δ is shown in blue mesh. The radius of the electron density map was set to 3.5 Å around the glycerol molecules.
